# Breastfeeding importance and its therapeutic potential against SARS‐CoV‐2

**DOI:** 10.14814/phy2.14744

**Published:** 2021-02-13

**Authors:** Aline Vasques da Costa, Carolina Purcell Goes, Patrícia Gama

**Affiliations:** ^1^ Department of Cell and Developmental Biology Institute of Biomedical Sciences University of São Paulo (USP) – São Paulo São Paulo Brazil

**Keywords:** breastfeeding, coronavirus, COVID‐19, diarrhea, milk, SARS

## Abstract

During postnatal development, colostrum and breastmilk are sequentially the first sources of nutrition with protein components and bioactive molecules that confer protection and immunostimulatory function to the gut. Caseins, whey proteins, secretory immunoglobulin A (sIgA), mucins, tryptophan, and growth factors are among milk‐borne elements that are directly important in the control of mucosa development and protection. Consequently, breastfeeding is associated with the low incidence of gastrointestinal inflammation and with the decrease in respiratory diseases during postnatal period. The novel coronavirus (SARS‐CoV‐2) binds to angiotensin II‐converting enzyme (ACE2) on the cell membrane, allowing virus entrance, replication, and host commitment. ACE2 is expressed by different cell types, which include ciliated cells in the lungs and enterocytes in the intestine. Such cells are highly active in metabolism, as they internalize molecules to be processed and used by the organism. The disruption of ACE2 impairs leads to intestinal inflammation and decreased synthesis of serotonin, affecting motility. By reviewing the effects of SARS‐CoV‐2 in the gastrointestinal and respiratory tracts in infants, and gut responses to breastfeeding interruption, we suggest that it is important to maintain breastfeeding during SARS‐CoV‐2 infection, as it might be essential to protect newborns from gastrointestinal‐associated disorders and relieve disease symptoms.

## Graphical abstract

We currently reviewed the effects of SARS‐CoV‐2 in the gastrointestinal and respiratory tracts in infants and discussed gut responses to breastfeeding interruption, and we suggest that it is important to maintain breastfeeding during SARS‐CoV‐2 infection, as it might be essential to protect newborns from gastrointestinal‐associated disorders and relieve disease symptoms.

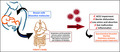

## 1 INTRODUCTION

The novel coronavirus (SARS‐CoV‐2) infection can cause pneumonia, diarrhea, multi‐organ failure, and death. Although newborn babies seem to have less severe clinical symptoms when compared to other age groups, both the cause for such low sensibility and the potential harm of this novel disease to them remain largely unknown. In terms of mechanism, the virus enters human cells after binding its spike glycoprotein (S protein) to the angiotensin II‐converting enzyme (ACE2) receptor located on the cell membrane, and then the spike protein is primed by transmembrane serine protease 2 (TMPRSS2) (Guo et al., 2020; Hoffmann et al., 2020; Neurath, 2020). This process consequently allows the virus entry, replication, and host commitment. The presence of ACE2 and TMPRSS2 proteins in several organs facilitates the infection occurrence (Hoffmann et al., 2020), which can lead to secondary effects as stroke, heart failure, muscle pain, diarrhea, and anosmia, in addition to respiratory syndrome (Grant et al., 2020).

Specifically, for gastrointestinal (GI) and respiratory infections, exclusive breastfeeding is important to control the increase of virulence, reducing the associated morbidity (Duijts et al., 2010). Many breastmilk components have antimicrobial properties, protect the newborn, reduce the infection rate, and/or alleviate the symptoms (Boix‐Amorós et al., 2019). Because of that, since the beginning of COVID‐19 pandemic in 2019, the World Health Organization (WHO) and the United Nations Children's Fund (UNICEF) gave special attention to pregnant women and newborns regarding the vertical or horizontal transmissions (Di Mascio et al., 2020; Mahyuddin et al., 2020). Thus, due the importance of exclusive breastfeeding in the general protection of infants, and because breastfeeding interruption can modulate the development of GI mucosa and immune system, the current review discusses the therapeutic potential role of breastmilk elements and the relevance of their investigation during COVID‐19 infection.

## 2 SARS‐COV‐2 AND GUT INFECTION

In the human body, SARS‐CoV‐2 binds to ACE2 receptor, which is highly expressed in the oral cavity and in several organs from GI and respiratory tracts, heart, and kidney. In the GI tract, ACE2 is expressed in the brush border of differentiated enterocytes, and diarrhea is the main response in part of the COVID‐19‐stricken patients (Cholankeril et al., 2020; Li et al., 2020; Xu, Zhong, et al., 2020).

Recent studies used scRNA‐seq to demonstrate the co‐expression of ACE2 and TMPRSS2 in the enterocytes in the gut of humans and primates (Hoffmann et al., 2020; Zhang et al., 2020; Ziegler et al., 2020). TMPRSS2 acts on the cleavage of virus S protein in the cell membrane, allowing the release of a viral peptide that is necessary for membrane fusion (Hoffmann et al., 2020). Thus, the co‐expression of ACE2 and TMPRSS2 is critical to the entry of SARS‐CoV‐2 into the host cell, suggesting that the respiratory and enteric symptoms of COVID‐19 are associated with the invasion of SARS‐CoV‐2. Lamers et al. (2020) demonstrated that in human small intestinal organoids (hSIOs) the virus targets progenitor cells and a large number of enterocytes, leading to apoptosis. However, this condition seems to be independent of ACE2 expression, as low levels of ACE2 mRNA were detected in infected enterocytes. Moreover, despite the high viral replication, SARS‐CoV‐2 infection did not induce cell death of Caco‐2 cells (Chu et al., 2020). Similarly, in organoids from human airways, SARS‐CoV‐2 was able to infect the ciliated cells, without promoting cell death (Chu et al., 2020; Lamers et al., 2020). Additionally, ACE2 knockout mice present increased inflammatory reaction, disruption of intestinal epithelial barrier, enhanced infiltration of inflammatory cells, and severe diarrhea (Hashimoto et al., 2012).

Diarrhea is a frequent symptom in coronavirus‐induced diseases such as *Severe Acute Respiratory Syndrome* (SARS) and *Middle East Respiratory Syndrome* (MERS), to which the incidence reaches up to 73%, and it is also the most important inducer of infantile death (Turin & Ochoa, 2014; Wong et al., 2020). However, the SARS‐CoV virus can replicate better in the intestinal cells when compared to SARS‐CoV‐2, indicating the reason why diarrhea is more usual in CoV infections (Chu et al., 2020). A meta‐analysis review with 24,412 adult patients from nine countries showed that the prevalence of respiratory and GI symptoms in adults was 23% and 16%, respectively (Grant et al., 2020). Pneumonia was present in 91% of the cases. When these same symptoms were observed in children, their frequencies in respiratory and GI tracts were 1–11% and 4–7%, respectively, representing 2.5‐fold less than the rate of infected adults (Ding et al., 2020; Mantovani et al., 2020; Wang et al., 2020). Moreover, only 8% of neonates and babies under 1‐year old had severe complications, presenting both respiratory and GI symptoms (Ding et al., 2020). Interestingly, the number of asymptomatic children was 19.6%, and pneumonia was found in 60% of the cases (Hoang et al., 2020). These observations confirm that the prevalence of COVID‐19 is higher in adults than in children.

## 3 BREASTMILK COMPOSITION AND ITS ANTIMICROBIAL ROLE

Human milk contains proteins and amino acids, ions, microorganisms, and other molecules that provide a wide range of biological activities and act to induce gut maturation and immunostimulatory functions, protecting against infectious diseases (Table 1) (Boix‐Amorós et al., 2019).

**TABLE 1:** Main bioactive molecules present in human breastmilk and their benefits (Feng et al., 2016; Lönnerdal et al., 2017; Peterson et al., 1998; Rangel et al., 2016; Wheeler et al., 2007)

Milk component	Benefits/function	mg/mL
Mucin	Mucosal barrier protection, antimicrobial action, immune system modulation	1.6
Lactalbumin	Antimicrobial action, immune system modulation	2.6
Lactadherin	Immune system modulation, inflammatory response	0.1
Casein	anti‐inflammatory role and mucosal protection	3.6
Immunoglobulins	Immune system modulation	1.1
Lactoferrin	Antimicrobial and antiparasitic action, promotes microbial homeostasis.	1.4
Amino acids	Antimicrobial action, microbial homeostasis, immune system modulation, tissue maturation	2.0

Among the protective milk‐borne molecules are caseins, whey proteins, tryptophan, lactadherin, mucin 1, lactoferrin, α‐lactalbumin, and secretory immunoglobulin A (sIgA) (Goldman, 1993; Roager & Licht, 2018). These elements are also involved in the development of the immune system and contribute to infants’ defense against both bacterial (*S*. *pneumoniae*) and viral pathogens (rotavirus, *influenza*, Ebola, cytomegalovirus, dengue virus, and respiratory syncytial virus) (Goldman, 1993; Lönnerdal, 2003; Pribylova et al., 2012; Santos et al., 2013; Walker & Meng, 2020).

### 3.1 Caseins

Caseins comprise a family of αs1, αs2, β, and κ proteins. Because of their hydrophobic behavior, they tend to form micelle complexes in the watery milk phase (Müller‐Buschbaum et al., 2007). The κ type of casein is the only calcium‐insensitive one, stabilizing the calcium and phosphorus binding of αs1‐, αs2‐, and β‐caseins (Müller‐Buschbaum et al., 2007). Upon digestion, casein proteins provide immunomodulatory peptides that are associated with lymphocyte proliferation, B‐ and T‐cell activation, macrophage phagocytic activity, and anti‐inflammatory role through reduction of cytokine expression (Daddaoua et al., 2005; Kayser & Meisel, 1996; Müller‐Buschbaum et al., 2007). For instance, a human κ‐casein‐derived fragment is able to induce hemagglutination of red blood cells and inhibition of *influenza* virus infection (Kawasaki et al., 1993). Similarly, another fragment confers resistance to *Klebsiella pneumoniae* in mice treated intravenously (Parker et al., 1984), inhibits adhesion to gastric cells, and promotes mucosal protection against infection with *Helicobacter pylori* (Strömqvist et al., 1995).

### 3.2 Whey proteins

In colostrum, non‐immunoglobulin components such as mucin and lactadherin are components of the membrane in the milk fat globule, and both play important roles in infant protection. Lactadherin is a membrane‐associated cell adhesion molecule that prevents symptomatic rotavirus infection by inhibiting pathogen binding, inducing IL‐10 and TGF‐β release from regulatory T cells, besides promoting intestinal dendritic cell development. Furthermore, lactadherin enhances macrophage phagocytic activity of apoptotic cells and improves the inflammatory response induced by NF‐kB and by mitogen‐activated protein kinase (He et al., 2016).

Among the many glycoproteins of human milk, lactoferrin is found mainly in the colostrum. Lactoferrin presents antibacterial and antiparasitic activities through inhibition of microbial adhesion, growth, and biofilm formation (Berlutti et al., 2011). It also regulates proliferation and differentiation of intestinal epithelial cells, limiting pathogen colonization of the intestinal tract (Arnold et al., 1980; Brock, 1980). Moreover, lactoferrin has an important function in the generation of an environment for the growth of beneficial bacteria in the gut, which protects against infection and inflammation by reducing the production of inflammatory cytokine at local sites (Pammi & Abrams, 2015). Evidence demonstrated the potential antiviral activity of lactoferrin combined with other whey proteins in the prevention and/or in the treatment of common cold viruses, and in the reduction of risk of sepsis in GI and respiratory tracts (Berlutti et al., 2011; Manzoni et al., 2009; Vitetta et al., 2013). One of the mechanisms involved in the prevention of virus entry into the host cell occurs through the blockage of cellular receptors, and in that way, heparin sulfate glycosaminoglycans (HSGs) interact with lactoferrin. Such system is used by coronaviruses, and the interaction of lactoferrin‐spike protein or spike‐HSG blocks SARS‐CoV cell infection (Lang et al., 2011). The direct binding to virus particles was also described for other respiratory syncytial viruses. Accordingly, lactoferrin interacts with the virus F protein and inhibits the entry into the cell and virus replication (Sano et al., 2003). In the GI tract, similar results were reported for rotavirus (Grover et al., 1997).

Besides lactoferrin and immunoglobulins, α‐lactalbumin is one of the most predominant proteins in human milk whey and it constitutes approximately 36% of milk total amount (Layman et al., 2018). α‐Lactalbumin is proteolyzed in many active peptides, that exert antibacterial function and stimulate lymphocyte proliferation and phagocytic activity in macrophages (Floris et al., 2003; Jaziri et al., 1992; Kayser & Meisel, 1996; Pellegrini et al., 1999). Whey is also an important source of other peptides and essential amino acids, including tryptophan, which contributes to infant nutrition and development (Layman et al., 2018).

In early life, breastmilk is the main source of tryptophan (Liu & Newburg, 2013), which is an essential amino acid precursor of several metabolites, such as serotonin and niacin (O’Mahony et al., 2015). Tryptophan controls the expression of small intestinal antimicrobial peptides, acts in the regulation of GI tract motility, in the microbiota homeostasis, and as anti‐inflammatory agent through aryl hydrocarbon receptors (AHR) (Borges et al., 2017; Hashimoto et al., 2012; Metidji et al., 2018; Singer et al., 2012). In addition, nicotinamide and dietary tryptophan appear to exert effects on colitis and diarrhea prevention via SLC6A19/B^0^AT1‐ACE2/mTOR‐related pathway, and on the regulation of intestinal cell proliferation, differentiation, migration, and cytoskeletal reorganization (Cole‐Jeffrey et al., 2015; Hashimoto et al., 2012; Kowalczuk et al., 2008; Wu, 2009). Interestingly, the absence or tryptophan deficiency, due to reduced absorption or to low protein diets, is related to disorders such as inflammatory bowel disease (IBD), irritable bowel syndrome, and Hartnup disorder (Taleb, 2019).

In cohort studies, infants fed exclusively with breastmilk were less susceptible to necrotizing enterocolitis (NEC) (Repa et al., 2015), presenting reduction of 32% to 80% in the number of cases (Lin et al., 2005; Repa et al., 2015). The combination of probiotics and breastmilk intake was protective against NEC, whereas for infants that were exclusive formula‐fed, it was ineffective. This response was due to the generation of indole‐3‐lactic (ILA), a breastmilk‐tryptophan metabolite provided by microbes, such as *Bifidobacterium*, in which proliferation is stimulated by the ingestion of breastmilk (Gregory et al., 2016; Jost et al., 2012; Meng et al., 2020). ILA was shown to be effective in reducing inflammation in a human NEC cell line and in immature enterocytes by interacting with AHR to prevent interleukin‐8 (IL‐8) expression (Meng et al., 2020). As aforementioned, the low levels of tryptophan lead to the decrease in the expression of antimicrobial peptides, resulting in a higher microbiome diversity and tissue inflammation (Hashimoto et al., 2012), and formula‐fed infants present this same pattern (Bäckhed et al., 2015). Other disorders such as colitis and Crohn's disease have been also related to the low tryptophan intake in humans (Beeken, 1976; Clayton et al., 1991). Besides tryptophan, glutamate and glutamine are also associated with protection against infections in early life (van Sadelhoff et al., 2020). Other authors suggest a possible link between COVID‐19 and gut dysbiosis due to the presence of GI bacteria in the lungs of COVID‐19‐positive patients and the onset of SARS (Dhar & Mohanty, 2020; He, Ren, et al., 2020; He et al., 2020). In this sense, as tryptophan regulates the gut microbiome, breastfeeding could enhance SARS‐CoV‐2 infection resistance, reducing the impact on the gut microbiome and on COVID‐19 severity. Accordingly, due to its importance to early development, the WHO recommendation is to a minimum intake of 8.5 mg/kg body weight of tryptophan until 6 months old (WHO, 2007), and this dose can be fully provided through breastmilk, especially in colostrum (O’Rourke et al., 2018).

Among milk proteins, sIgA is the predominant immunoglobulin, representing 25% of total milk proteins and 90% of the antibodies present in human milk (Hurley & Theil, 2011). It is resistant to proteolysis (Demers‐Mathieu et al., 2018) and can be transferred to the infant via the entero‐mammary pathway. sIgA‐producing B cells originate in the intestine, and through lymph and blood, they migrate to different glands, including the mammary gland. sIgA crosses gland cells through transcytosis to the lumen and becomes part of the milk content, conferring the neonate and the infant protection against respiratory and intestinal infections (Telemo & Hanson, 1996). In newborn pigs, a protein‐deficient diet reduces the amount of sIgA generated in response to a viral agent. The low protein intake and, consequently, the low absorption of tryptophan via ACE2 impair immune response, and induce deregulation of microbial homeostasis and intestinal inflammation (Fischer et al., 2017). Thus, breastfeeding interruption leads to low protein intake and may affect the newborn nutrition, development, and infection response.

A recent study reported the presence of sIgAs against the SARS‐CoV‐2 spike protein in 100% (15) of mothers recovered from COVID‐19, and 80% presented reactivity to receptor‐binding domain of the spike protein (Fox et al., 2020). Additionally, the same study demonstrated the presence of IgG and/or IgM in eight of 12 patients postinfection, but IgA and IgG were found at highest levels. Other study also detected IgA reactivity to SARS‐CoV‐2 in 97% (39 women) of breast milk samples (Demers‐Mathieu et al., 2020) As these antibodies persist in breastmilk in high levels for at least 7 months postpartum (Rechtman et al., 2002), they may be used as a therapy to prevent SARS‐CoV‐2 infection or COVID‐19 symptoms in the first year of life. Moreover, the large‐scale production of these antibodies may help the recovery in severe cases.

### 3.3 Effects of breastfeeding interruption on gut development and protection

During postnatal development, the period of exclusive breastfeeding is followed by a gradual transition to solid food intake, which characterizes weaning. Early weaning (EW) represents the abrupt interruption of exclusive breastfeeding to food intake before 6 months old, regardless the reason for such break. Interestingly, different studies reported that exclusive breastfeeding (no supplementation of other formula, milk, solids, or fluids), followed by partial breastfeeding (breastmilk with or without other liquids or solids) during 6 months or more were associated with a lower risk of GI tract infection when compared with infants that were EW before 6 months (Duijts et al., 2010; Kramer et al., 2003; Quigley et al., 2007). Accordingly, a cohort study with 815 mothers and their infants showed that 207 infants under 3 years old were hospitalized due to severe infections. In addition, the hospitalization rates decreased as breastfeeding duration was extended for a longer period, more specifically 5% for every extra month of breastfeeding (Christensen et al., 2020). Interestingly, the babies exclusively breastfed for 4 months or more showed 50% lower infection rates, and in the first year of life, the association between breastfeeding duration and hospitalization was stronger than the other correlations analyzed, suggesting that extended breastfeeding periods might help preventing infections in early life.

During the postnatal period, breastfeeding is the first source of bioactive agents, which are important to establish GI function and immune ontogeny (Goldman, 1993). EW and the change to solid food affect the development of pulmonary and GI tracts and interfere in cell proliferation and differentiation. In the gastric mucosa, EW stimulates cell proliferation through MAPK signaling (Cummins & Thompson, 2002; Osaki et al., 2011) and it interferes in cell differentiation (Zulian et al., 2017; Teles Silva et al., 2019). Similarly, we demonstrated that EW also reduces the differentiation of intestinal goblet cells in rats (da Costa et al., 2019). These cells produce mucins that are the glycoproteins that confer physical, biological, and mechanical protection to epithelial cells from the oral cavity to colon and other tissues (Linden et al., 2008). Mucins constitute an effective barrier against viral infections (e.g., coronaviruses, *influenza*, enteroviruses) due to the binding of their sialic acids (Alexander & Dimock, 2002; Couceiro et al., 1993; Matrosovich & Klenk, 2003; Schwegmann et al., 2001). However, as breastfeeding absence affects goblet cells and, consequently, mucin expression, the maternal separation and milk‐formula substitution could be an open gate to microbial/viral infections such as SARS‐CoV‐2. Accordingly, intestinal damage, infection, and the number of apoptotic cells were increased in mucin 1‐knockout mice, confirming its protective function in mucosal barrier (McAuley et al., 2007). Interestingly, MUC1 is also a milk‐borne molecule, and some studies demonstrated its antimicrobial action against rotavirus, *H*. *pylori*, *C*. *jejuni*, *Staphylococcus aureus*, *Salmonella enterica*, and *Pseudomonas aeruginosa*, reducing also the damage induced in the epithelium (Liu et al., 2012; Lu et al., 2006; McAuley et al., 2007; McGuckin et al., 2007; Parker et al., 2010; Peterson et al., 2001; Santos et al., 2013; Yolken et al., 1992). Other intestinal mucins such as MUC5AC and MUC6 are associated with the modulation of inflammatory response (Reis et al., 1999; Rokhsefat et al., 2016), which contributes to a lower risk of morbidity and, consequently, infant mortality. Thus, the expression of mucins becomes indispensable for development, maturation, and intestinal protection.

Immunologically, the traffic of maternal immune cells from breastmilk to the infant mucosal surfaces and other tissues contributes to the appropriate selection of commensal bacterial populations in the GI tract and the final maturation of the immune system (Howson et al., 2015; Jost et al., 2014). Several studies demonstrated the positive correlation between the concentration of milk TGF‐β and infant immunoglobulin production. The presence of TGF‐β in breastmilk attenuates the inflammatory response to cytokines IL‐1β and decreases neonatal diseases, including respiratory impairment and allergy (Oddy & Rosales, 2010; Ogawa et al., 2004). In EW piglets, the immune system response induces the high expression of pro‐inflammatory molecules, such as TNF‐α, IL‐6 and IL‐1β, and it increases immune cell infiltration in the small intestine (McLamb et al., 2013; Orgeur et al., 2001; Pié et al., 2004), suggesting that EW animals may develop a pro‐inflammatory cascade. Similar responses were reported in infants with SARS‐CoV‐2 infection, indicating that both COVID‐19 and breastfeeding interruption can contribute to gut inflammation (Xu, Li, et al., 2020). Conversely, breastfeeding maintenance could reduce the inflammatory response to SARS‐CoV‐2 and other infections.

## 4 DISCUSSION

In the current review, we considered the data from reports on GI infection, inflammatory conditions, and the effects of breastfeeding on respiratory and GI tracts, and we discussed the strategies used by SARS‐CoV‐2 in gut cells and the importance of breastmilk molecules.

**FIGURE 1:**
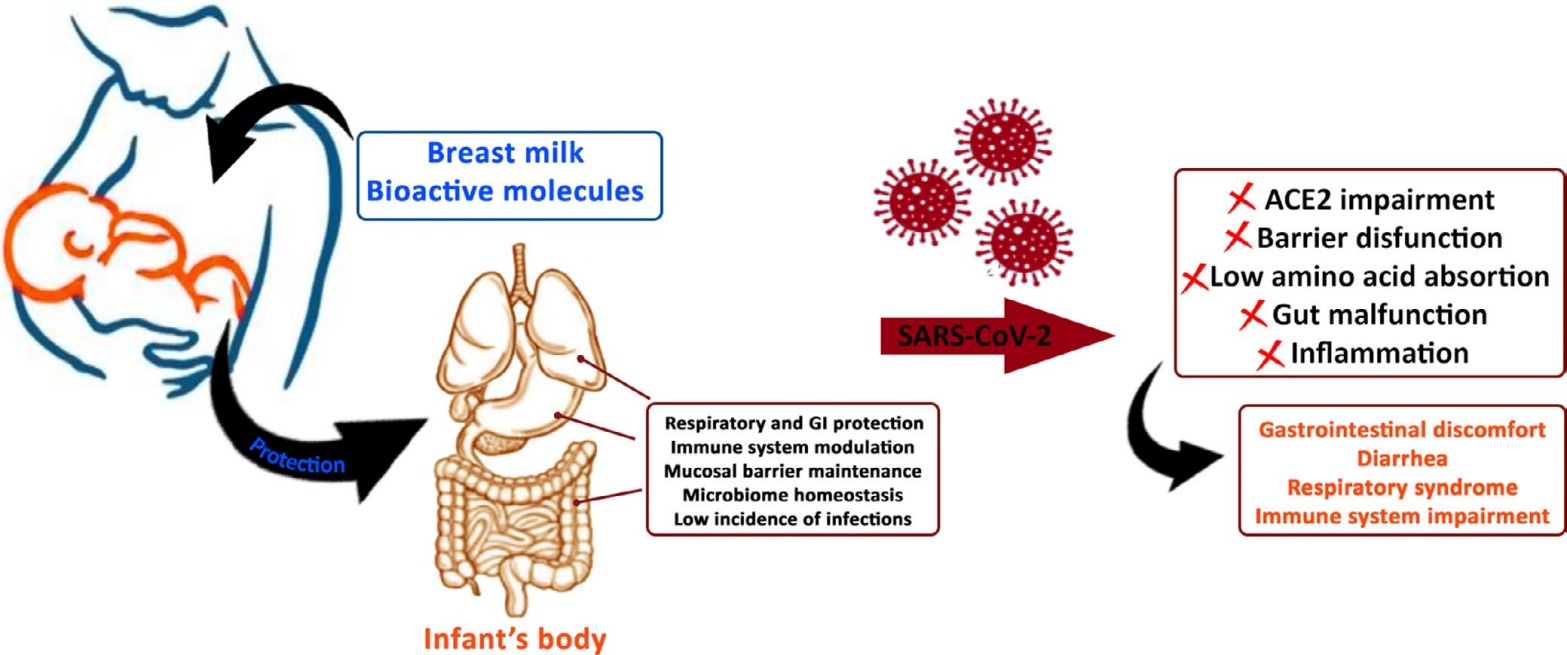
Protective effects of breastfeeding in the intestine. Milk‐borne molecules and their metabolites reach the intestinal epithelium, and promote the development and maturation of GI, immune, and respiratory tracts. During SARS‐CoV‐2, homeostasis mechanisms are affected, such as ACE2 function and mucosal barrier, disturbing tissue functions, which contribute to gut malfunction, diarrhea, pneumonia, and systemic inflammation

The association of breastfeeding with low incidence of GI manifestations as diarrhea, and respiratory infections suggest that breastmilk is essential to deliver the necessary elements for prevention of frequent intestinal inflammation, decrease respiratory diseases during postnatal period, and to support infant immune development (Figure 1). Additionally, the breastmilk microbiome is essential in the composition of infant's intestinal microbiome diversity, contributing to the gut–lung axis functions and immune system homeostasis (He, Ren, et al., 2020). Thus, both the microbial inhibitory effects derived from the components of breastmilk (Lang et al., 2011) and the low incidence of co‐infections related to severe cases of COVID‐19 prompted us to suggest that breastmilk may have a protective role in early life, either directly against SARS‐CoV‐2 infection or indirectly by relieving COVID‐19 symptoms (Dong, Mo, et al., 2020; Hoang et al., 2020).

The limited evidence of the presence of SARS‐CoV‐2 in breastmilk that was reported in only 11 mothers at postpartum period (Centeno‐Tablante et al., 2021; Chambers et al., 2020; Costa et al., 2020; Groß et al., 2020; Tam et al., 2021) indicates that studies with the novel coronavirus still need deep investigation and depend on the validation of analytical methods for human milk to allow the discussion of the vertical transmission (Lackey et al., 2020). In addition, there are also no reports demonstrating the presence of other coronaviruses such as SARS‐CoV and MERS in human milk (Schwartz & Graham, 2020).

The evens and odds of breastfeeding interruption should be carefully evaluated in cases of infected mothers as well. Since antibodies (IgAs, IgGs, and IgMs) and other molecules are acquired through breastfeeding in early life, breastmilk may have a therapeutic potential in relieving respiratory and GI symptoms and, possibly, other systemic responses (Dong, Chi, et al., 2020; Fox et al., 2020). However, it is still unknown whether those antibodies can neutralize SARS‐CoV‐2. Davanzo (2020) highlights the importance of considering the beneficial effects of breastfeeding, whereas Jing et al. (2020) suggest that it should be suspended for 14 days and babies fed with artificial milk. However, the consequences of maternal separation within the first days postnatal reflect in development, immunity, and behavior (Carlyle et al., 2012).

Importantly, we consider that future studies should include the meta‐analysis of public data associated with genomic and proteomic studies to evaluate the association among infection, social conditions, COVID‐19 progression, breastfeeding maintenance, and the expression of genes and proteins that characterize the regular development and growth of GI tract. Moreover, we believe that experimental models could be used in the investigation of the roles of milk‐borne bioactive molecules against SARS‐CoV‐2 and their therapeutic potential.

Therefore, as the findings herein reviewed suggest that breastfeeding benefits (Table 1) overlap with the odds and there are not much data reporting the presence of SARS‐COV‐2 in breastmilk samples, we support the maintenance of breastfeeding and reinforce WHO guidelines to safely breastfeed the newborns (WHO, 2020).

## COMPETING INTERESTS

The authors declare that the research was conducted in the absence of any commercial or financial relationships that could be construed as a potential conflict of interest.
